# Newly diagnosed papillary craniopharyngioma with *BRAF V600E* mutation treated with single-agent selective BRAF inhibitor dabrafenib: a case report

**DOI:** 10.18632/oncotarget.27203

**Published:** 2019-10-15

**Authors:** Mayank Rao, Meenakshi Bhattacharjee, Scott Shepard, Sigmund Hsu

**Affiliations:** ^1^The Vivian L. Smith Department of Neurosurgery, McGovern Medical School, The University of Texas Health Science Center at Houston, Houston, TX 77030, USA; ^2^Department of Pathology and Laboratory Medicine, McGovern Medical School, The University of Texas Health Science Center at Houston, Houston, TX 77030, USA; ^3^Department of Neurosurgery, Temple University School of Medicine, Philadelphia, PA 19140, USA

**Keywords:** papillary, craniopharyngioma, *BRAF V600E*, dabrafenib, neuro-oncology

## Abstract

We report a case of a patient with newly diagnosed, locally extensive and cystic, suprasellar papillary craniopharyngioma successfully treated with single-agent Dabrafenib. The patient was symptomatic with gait imbalance with falls, lethargic episodes, fatigue and incontinence. Diagnostic imaging demonstrated a cystic suprasellar tumor extending into the third ventricle causing obstructive hydrocephalus. The tumor was partially debulked, and bilateral shunts were placed. NGS sequencing demonstrated *BRAF V600E* mutation, and the patient was prescribed dual agent Dabrafenib and Trametinib. However, due to insurance denial for Trametinib, he only received single-agent Dabrafenib (150mg BID). The treatment resulted in a major response (over two years), including reduction of the tumor cyst, and improvement of the clinical symptoms. No adverse events have been reported. The patient continues on Dabrafenib (150 mg BID) with a steady reduction in tumor size, and improvement in cognitive function leading to independent living.

## INTRODUCTION

Craniopharyngiomas are uncommon primary brain epithelial tumors, characterized by a low proliferation rate, symptomatic due to mass effect and local infiltration of the surrounding tissues. They are typically located in the sellar region of the brain and infiltrate adjacent areas such as optic nerves, pituitary gland, hypothalamus and the brainstem. Although they are considered histologically benign, these tumors create significant treatment challenges. Standard treatment includes surgical resection (trans-sphenoidal or trans-cranial approach) and external beam radiotherapy for residual or recurrent tumor. Common symptoms observed as a result of tumor growth or treatment include visual loss, pan-hypopituitarism, cognitive deficits, personality changes, hyperphagia and morbid obesity [1]. Craniopharyngiomas occur in two histological subtypes, adamantinomatous and papillary, with similar presentations and response to standard surgical treatments. There are no recognized targeted or cytotoxic treatments available for adamantinomatous craniopharyngioma [2–3]. There is a developing new standard of targeted therapy of papillary craniopharyngioma.

Brastianos reported that 95% of papillary craniopharyngiomas harbor the *BRAF V600E* mutation [3]. Functionally, this mutation activates the mitogen-activated protein kinase (MAPK) pathway. The MAPK pathway is a major intracellular signal transduction pathway that is responsible for cellular proliferation, gene expression, differentiation, mitosis, cell survival, and apoptosis [4]. Experimentally, in vitro, the mutation is a driver mutation, and when *BRAF V600E* was ectopically expressed in fibroblast cell lines, it caused hyper stimulation of the MAPK cascade and malignant transformation [2, 4]. Brastianos reported a dramatic response of a BRAF mutant recurrent papillary craniopharyngioma in 2016 to targeted therapy with combination Dabrafenib and Trametinib [7]. Moreover, we performed a literature search and did not identify any papillary craniopharyngioma *BRAF V600E* non-responders related case reports or research articles.

We report the successful use of single-agent Dabrafenib in a newly diagnosed patient with extensive residual papillary craniopharyngioma, positive for the *BRAF V600E* mutation (Table 1).

**Table 1:** Summary of papillary craniopharyngioma targeted therapy case reports

Newly diagnosed or recurrent PCP	Treatment with *BRAF V600E* +/- MEK inhibitor	Treatment duration	% reduction in tumor size (volume)	Toxicity	Reference
Recurrent	Dabrafenib (150 mg BID) and Trametinib (2 mg BID)	1.1 months (35 days)	85%	Low grade fever	Brastianos, et al. (2015) [7]
Recurrent	Dabrafenib (150 mg BID) and Trametinib (2 mg QD)	7 months	>75%	Low grade fever	Odia, et al. (2017) [8]
Recurrent	Dabrafenib (150 mg BID) and Trametinib (2 mg QD)	3.4 months (15 weeks)	91%	Low grade fever	Gudjonsson, et al. (2017) [9]
Recurrent	Vemurafenib (960mg BID)	3 months	Near complete resolution of PCP	NA	Aylwin, et al. (2016) [10]
Recurrent	Dabrafenib (150 mg BID, then dose reduced for toxicity to 225mg QD)	9 months	Marked reduction (>50%) after 9 months of therapy and patient remained stable post 1 year of Dabrafenib cessation	Joint pain	Uhm, et al. (2018) [11]

## CASE PRESENTATION

A 35-y/o Caucasian male presented with progressive short-term memory loss, and chronic headaches associated with daily nausea and vomiting. His family further reported gait imbalance with falls, episodes of lethargy, fatigue and incontinence. The patient’s past medical history and social history was negative. At OSH, the patient was diagnosed as hypotensive and bradycardic (HR 30s) and was given dopamine before being transferred out. On admission, patient HR was 50-60 with systolic BP at 90 mm Hg.

MRI brain with and without contrast showed a third ventricular mass with obstruction of the foramen of Monroe resulting in hydrocephalus. The patient required placement of bilateral shunts for hydrocephalus and craniotomy for resection. The tumor was diagnosed as papillary craniopharyngioma, WHO Grade I. Post craniotomy MRI brain confirmed that the patient had residual tumor close to the fornix. The patient required desmopressin (DDAVP), levothyroxine, and dexamethasone. Next-generation sequencing (Foundation One) identified the *BRAF V600E* genomic alteration.

**Figure 1:**
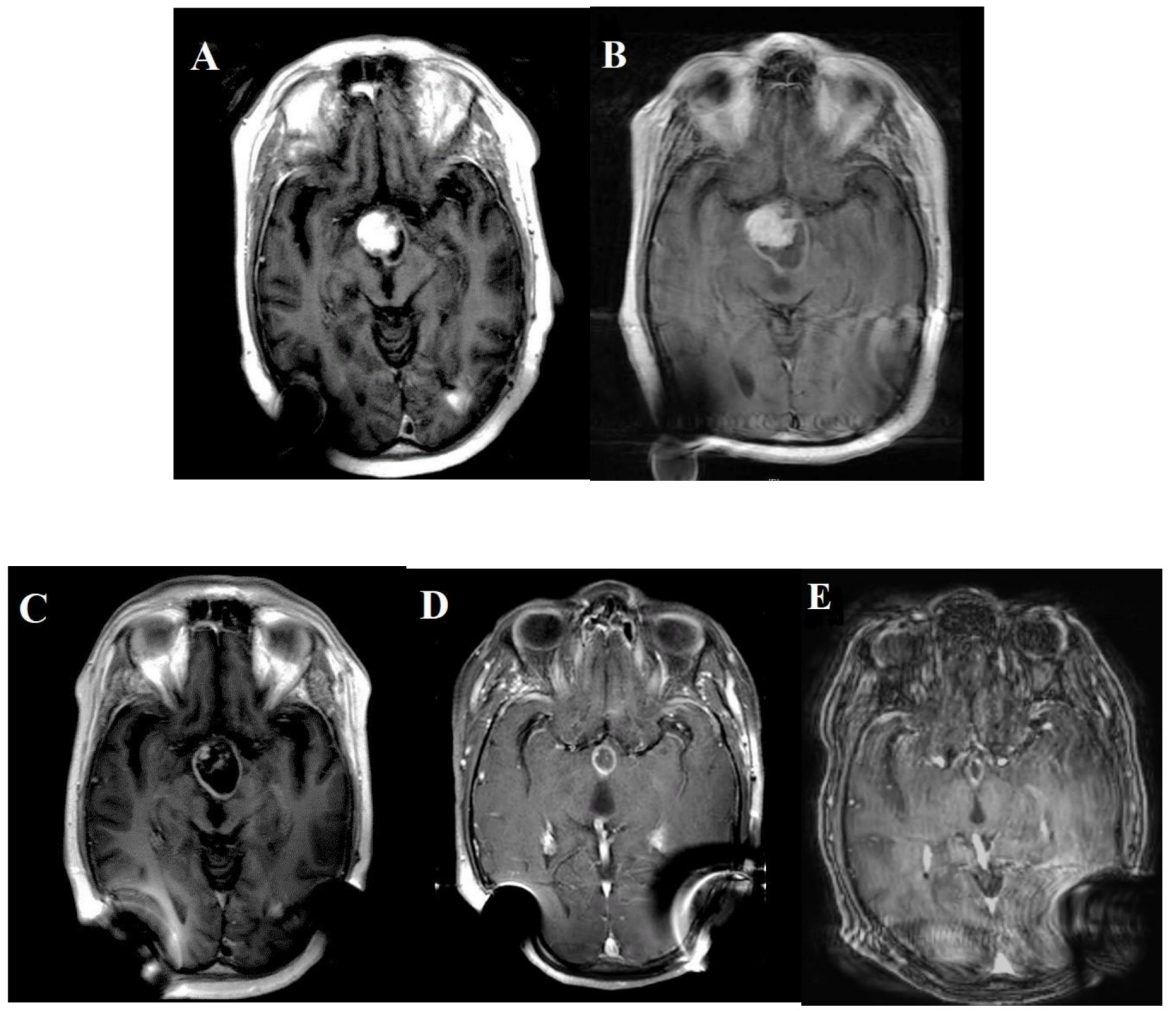
MRI brain before BRAF inhibitor was introduced (both A & B) B/L posterior distortions are the result of B/L shunts for hydrocephalus (left) and (right) respectively. MRI on the right **(B)** showed an increase in the size of the residual suprasellar craniopharyngioma (48 x 53 x 29 mm) predominantly due to an increase in the size of the cystic component. After BRAF inhibitor was introduced, MRI brain was done at two-month intervals. MRI in the bottom left **(C)** shows partial response with a significant reduction in the solid tumor. MRI in the bottom middle **(D)** was a year after treatment. It shows the patient's solid internal nodular enhancement, and his cyst completely resolved as compared to the original MRI **(A)** in the top left from February 2016. Starting May 2017, the MRIs were performed every six months. The right bottom MRI **(E)** continued shrinkage of the patient's suprasellar cystic lesion. It was two years after being on single-agent Dabrafenib.

Post-operatively, the patient’s MRI brain demonstrated residual suprasellar craniopharyngioma (45 x 39 x 25 mm) with multiple cystic components (Figure 1A). Efforts to obtain Dabrafenib and Trametinib were unsuccessful and repeat MRI brain showed an increase in the size of cystic components (48 x 53 x 29 mm) with stable nodular disease and resolution of his hydrocephalus (Figure 1B). Clinically, the patient continued to improve in strength but had a significant disability with his cognitive status. He continued to take HRT, and his dexamethasone dose was reduced to 0.5 mg PO QD.

On appeal, the insurance carrier denied the MEK inhibitor Trametinib treatment, but approved the single-agent Dabrafenib, which was initiated at 150mg BID. Two months after treatment, the patient’s family noted improved cognitive speed and balance. No skin, GI toxicity or seizures were reported. MRI brain demonstrated decreased size, enhancement and edema of the residual suprasellar craniopharyngioma (Figure 1C).

The patient’s progressive MRI brain examinations demonstrated a further interval decrease in size (Figure 1D). Clinically, the patient continued to slowly improve in his exercise tolerance and steroid myopathy. The dexamethasone dose was continued at 0.5 mg PO QD. The patient continued to have severe pan hypopituitarism, and continued his HRT treatment along with the addition of testosterone replacement therapy. Two years later, the MRI brain demonstrated the continued collapse of the patient’s cyst cavity and complete response of the solid internal nodular enhancement (Figure 1E). Clinically, the patient continued to improve steadily; he continued to require desmopressin at low doses and his short-term memory and cognitive processing speed improved. The patient was not formally assessed with neurocognitive testing but is able to live independently and resume driving and working.

## DISCUSSION

The discovery of activating *BRAF* mutation in melanoma and the development of targeted molecular therapy represented a major advance in the management of melanoma based on BREAK clinical trials [4]. In both papillary and adamantinomatous craniopharyngiomas, V600E and beta-catenin mutations (respectively) are not always present, hence the need for sequencing analysis [1–3]. In previous studies, it was reported that the treatment of metastatic melanoma harboring *BRAF V600E* mutation treated with Dabrafenib showed an effective response [4, 5]. In other reports, recurrent papillary craniopharyngioma with *BRAF V600E* mutation was treated with a combination of Dabrafenib and Trametinib therapy and showed an increase in the response rate [6, 7]. Due to these findings, the proposal to use single-agent Dabrafenib appeared a reasonable strategy. A recent report of using single-agent Dabrafenib for a recurrent craniopharyngioma rationalized its application further [9]. Our report of near complete and durable response to the use of Dabrafenib supports the pathogenic role of the *BRAF V600E* mutation in newly diagnosed papillary craniopharyngioma. It can also provide an additional option for patients intolerant to combination therapy with Dabrafenib and Vemurafenib.

### BRAF V600E mutation in papillary craniopharyngioma

Since craniopharyngiomas are suprasellar tumors, associated with significant morbidity and poor quality of life often follows local treatment [1–3, 7–9]. No standard chemotherapy exists, and when tumors recur after surgery, radiation treatment is effective, but not curative [7]. The V600E mutation activates the MEK-ERK pathway, and the mutation-specific *BRAF* inhibitor Dabrafenib, which targets mutated *BRAF*, is effective in malignant and metastatic melanoma, including the brain metastasis [4–6]. Brastianos identified 95% prevalence *BRAF V600E* mutation in the canonical series of papillary craniopharyngiomas [1–3]. Assessment of BRAF V600E mutation should be considered as the standard of care for craniopharyngiomas.

Currently, a phase II study of MEK (Cobimetinib 60mg QD) and BRAF (Vemurafenib 960mg BID) inhibitor is ongoing on *BRAF V600E* mutated papillary craniopharyngioma patients resected surgically (NCT03224767). The patients are divided into two cohorts: no prior treatment or received radiation therapy and progressive disease. We await efficacy results to guide multidisciplinary treatment planning for papillary craniopharyngioma patients.

If trials confirm the effective and durable response of papillary craniopharyngioma (PCP) to targeted therapy, it may be possible to reduce morbidity with biopsy, as opposed to resection. If NGS sequencing is validated for blood circulating cell-free tumor DNA “liquid biopsy”, for papillary craniopharyngioma, the need for tissue biopsy of the lesion may be unnecessary, and the patient could be empirically treated with BRAF/MEK inhibitor [1–3, 7].

### Dabrafenib as single agent treatment

Dabrafenib is a reversible, potent and selective *BRAF V600E* inhibitor [4]. In the treatment of malignant melanoma, (prior to the development of immune-oncology checkpoint inhibitors) it is regarded as less toxic, with a rapid mode of action, and prolongs progression-free survival (PFS) compared to chemotherapy [4–6]. The BREAK-MB clinical trial conducted for melanoma brain metastases with *BRAF V600E/K* suggested that Dabrafenib may be an effective adjunct for treatment of brain metastases (alongside surgery and radiotherapy) [4]. FDA has approved use of Dabrafenib in combination with Trametinib for the adjuvant treatment of melanoma patients with BRAF V600E/K mutations based on COMBI-AD trail [6]. The most common toxicity symptoms from clinical trials were rash, hyperkeratosis, fatigue, headache, arthralgia and pyrexia [4–6]. Pyrexia is specific toxicity seen with Dabrafenib that may respond to steroids [4]. Dabrafenib has a more favorable side effect profile as a single agent compared to the combination treatment of Dabrafenib and Trametinib, or single-agent Vemurafenib. We report no side effect of Dabrafenib in our patient with over two years of continuous use.

Brastianos further reported treatment of a 39-year-old male with multiple recurrent *BRAF V600E* craniopharyngioma using Dabrafenib (150mg PO bid) and Trametinib (2mg PO bid) [2, 7]. A blood-based *BRAF V600E* assay detected circulating *BRAF V600E* in the patient’s blood. After 35 days of treatment, tumor volume was reduced by 85% based on MRI scans [7]. The case reported the first exceptional therapeutic response to combined BRAF and MEK-targeted therapy in a multiply recurrent papillary craniopharyngioma with genetically confirmed *BRAF V600E* mutation. Both the solid and cystic portions of the tumor shrank rapidly. Recent case reports, using the same combination of BRAF and MEK inhibitors, showed similar results with more than 75% reduction in the size of papillary craniopharyngioma tumor [8, 9]. The course of action of using a combination of Dabrafenib/Trametinib therapy had low toxicity [7–9]. Genomic studies confirmed *BRAF V600E* mutation to be present in all tumor cells both before and after treatment, with the mutation also detectable in peripheral blood during treatment [7].

Similarly, Aylwin reported the response of a recurrent papillary craniopharyngioma with the *BRAF V600E* mutation to single-agent Vemurafenib (960mg PO BID), with a marked reduction in the size of the tumor, along with the resolution of the surrounding edema [10]. This tumor was exceptionally responsive to targeted treatment, with a near-complete radiological response after three months. However, when Vemurafenib was held, the tumor regrew in six weeks. Tumor growth was stabilized when Vemurafenib was re-administered, but eventually, the tumor progressed even with continued treatment [10].

Recently, an article reported the use of single-agent *BRAF V600E* Dabrafenib to treat recurrent papillary craniopharyngioma in a 52-year-old male patient [11]. Prior to initiation of Dabrafenib, the patient underwent standard of care radiation therapy. The initial dose was kept as 150mg BID, but was reduced to 225mg QD due to an adverse event (arthralgia) [11]. The reduction of tumor size and improvement of his clinical symptoms led to discontinuation of the Dabrafenib therapy after one year. The one-year follow-up, post-drug discontinuation, the subject reported being doing well both clinically and radiographically [11].

We report a clinically significant and durable near complete response of *BRAF V600E* in a newly diagnosed papillary craniopharyngioma to single-agent Dabrafenib. The patient is on single-agent *BRAF V600E* inhibitor (Dabrafenib 150mg BID) for two years and has been followed up both clinically and radiographically. The reduction in the size of the cystic cavity is especially compelling and our patient has continued to recover both endocrine and cognitive function. No adverse events such as pyrexia, skin or GI toxicity have been reported.

## CONCLUSIONS

Based on this case, single-agent treatment with Dabrafenib (150mg BID) is an option for papillary craniopharyngioma. Although the combination of Dabrafenib and Trametinib may have more rapid response, the combination also has increased toxicity so that single-agent BRAF inhibitor may be better tolerated. All papillary craniopharyngioma should undergo testing for *BRAF V600E* mutation before beginning treatment. In the future, we hope blood-based next-generation sequencing will be sensitive and specific enough for diagnosis and treatment. We believe the use of single-agent Dabrafenib is an option to treat newly diagnosed papillary craniopharyngioma with the *BRAF V600E* mutation, especially if patients are intolerant to combination BRAF and MEK inhibition.
