# Addendum: Newly diagnosed papillary craniopharyngioma with BRAF V600E mutation treated with single-agent selective BRAF inhibitor dabrafenib: a case report

**DOI:** 10.18632/oncotarget.28428

**Published:** 2023-05-10

**Authors:** Mayank Rao, Meenakshi Bhattacharjee, Scott Shepard, Sigmund Hsu

**Affiliations:** ^1^The Vivian L. Smith Department of Neurosurgery, McGovern Medical School, The University of Texas Health Science Center at Houston, Houston, TX 77030, USA; ^2^Department of Pathology and Laboratory Medicine, McGovern Medical School, The University of Texas Health Science Center at Houston, Houston, TX 77030, USA; ^3^Department of Neurosurgery, Temple University School of Medicine, Philadelphia, PA 19140, USA

**This article has an addendum:** Informed patient consent was obtained.

Original article: Oncotarget. 2019; 10:6038–6042. 6038-6042. https://doi.org/10.18632/oncotarget.27203

